# The aetiology of pharyngotonsillitis in primary health care: a prospective observational study

**DOI:** 10.1186/s12879-021-06665-9

**Published:** 2021-09-17

**Authors:** Jon Pallon, Mattias Rööst, Martin Sundqvist, Katarina Hedin

**Affiliations:** 1 Department of Clinical Sciences in Malmö, Family Medicine, Lund University, Malmö, Sweden; 2 Department of Research and Development, Region Kronoberg, Växjö, Sweden; 3 Department of Laboratory Medicine, Clinical Microbiology, Faculty of Medicine and Health, Örebro University, Örebro, Sweden; 4 Futurum, Region Jönköping County, and Department of Health, Medicine and Caring Sciences, Linköping University, Linköping, Sweden; 5 Department of Clinical Sciences, Malmö, Clinical Research Centre, Box 50332, 202 13 Malmö, Sweden

**Keywords:** Pharyngotonsillitis, Predictive values, Primary health care, Group A streptococci, Symptoms

## Abstract

**Background:**

Few studies on pharyngotonsillitis have examined the clinical presentation of different aetiologies where pathogens have been detected using molecular methods. We aimed to assess how well clinical signs and symptoms can predict (1) the presence or absence of a broad range of viruses and bacteria, and (2) reconsultations for a sore throat or a complication.

**Methods:**

In this descriptive observational prospective study in primary health care 220 patients aged 15–45 with suspected pharyngotonsillitis were sampled from nose, throat and blood and screened for 20 bacteria and viruses using polymerase chain reaction (PCR), culture and serology. Odds ratios (OR) and predictive values with 95% confidence intervals (CI) were used to show association between microbiological findings and clinical signs and symptoms. Patients were followed up after 3 months by reviewing electronic medical records.

**Results:**

Both cough and coryza were more common in patients with only viruses (67%) than in patients with only bacteria (21%) (p < 0.001), whereas tonsillar coating was more common in patients with only bacteria (53%) than in patients with only viruses (29%) (p = 0.006). Tonsillar coating (adjusted OR 6.0; 95% CI 2.5–14) and a lack of cough (adjusted OR 3.5; 95% CI 1.5–8.0) were significantly associated with *Streptococcus pyogenes* (group A streptococci; GAS) and with any bacterial finding. A Centor score of 3–4 had a positive predictive value of 49% (95% CI 42–57) for GAS and 66% (95% CI 57–74) for any bacterial findings. The use of rapid antigen detection test for GAS increased the positive predictive value for this group to 93%.

**Conclusions:**

Signs and symptoms, both single and combined, were insufficient to rule in GAS or other pathogens. However, both cough and coryza were useful to rule out GAS. The results support the clinical approach of restricting rapid antigen detection testing to patients with 3–4 Centor criteria. The low carriage rate of bacteria among asymptomatic controls implied that most detections in patients represented a true infection.

**Supplementary Information:**

The online version contains supplementary material available at 10.1186/s12879-021-06665-9.

## Background

Acute sore throat, or pharyngotonsillitis, is one of the most common reasons for consultation in primary health care [1]. Throat infections are most often of viral aetiology [2] but can also be caused by bacteria, of which *Streptococcus pyogenes* (group A streptococcus; GAS) is the most important and the only one to have a definitive indication for treatment in many guidelines, e.g. the Infectious Diseases Society of America [2] and The Sore Throat Guideline Group within the European Society for Clinical Microbiology and Infectious Diseases [3]. The clinical presentation of pharyngotonsillitis, however, overlaps broadly in GAS and non-GAS aetiology and individual signs and symptoms are not sufficient to discriminate between the two [4]. Attempts have therefore been made to group signs and symptoms into clinical scoring systems to increase the diagnostic accuracy [4–7]. The four item Centor score, invented in 1981 [8], is a well-calibrated and validated score [4, 5, 8] for detecting GAS in throat cultures, as is the newer FeverPAIN score [6]. Though easy to use, these scores only increase the positive predictive values to modest levels, especially in low-prevalence settings [7, 9], which is why several guidelines in North America and Europe recommend the addition of a rapid antigen detection test (RADT) for GAS [2, 3, 10]. In Sweden the Medical Products Agency recommends the use of such a test in patients with a Centor score of 3–4 (out of a maximum of 4 points), if they are thought to benefit from antibiotics, and to only prescribe antibiotics to patients who test positive [10].

Looking beyond GAS, there is some support for group C and G streptococci to present in a similar manner to group A [3, 11, 12]. The same goes for the anaerobic bacteria *Fusobacterium necrophorum*, most often detected in young adults with pharyngotonsillitis [13–15], though it has also been associated with a cough [14]. These alleged similarities between different bacteria [3, 11–15] has led some researchers to suggest that clinical scoring systems in fact predict the presence of bacteria, rather than GAS only [11–13]. For instance, the creators of the FeverPAIN score claim that their score detects both GAS and group C and G streptococci and recommend that treatment be guided by score rather than aetiology [12]; Centor et al. have argued that the Centor score predicts not only group A, but also group C and G streptococci [13]and *F. necrophorum* [13]; and Lindbaek et al. have also suggested that the Centor score predicts group C and G streptococci in addition to GAS [11]. Nevertheless, the studied pathogens in these papers have been restricted to a narrow range of bacteria [11–13], and there is still a lack of studies that investigate the clinical signs and symptoms of a broad range of bacteria and viruses using polymerase chain reaction (PCR) technique [3].

We previously published an aetiological prospective case–control study on young adults with pharyngotonsillitis in primary health care [14], with a subsequent 2-year follow-up study on the same patients and controls [16]. The present study was a reanalysis of these data sets, but with a shifted focus to a clinician’s perspective, examining how different signs and symptoms predict the presence or absence of various viruses and bacteria.

Our aims of this study were to describe how different signs and symptoms are associated with a wide range of aetiologies in pharyngotonsillitis, and to assess the association between the clinical presentation and return visits for a sore throat or for a complication within 30 days, or for a sore throat or tonsillectomy within three months. As we later discovered that 94% of the patients had been subjected to a RADT for GAS, we also aimed to describe both the performance of this test in our population and the underlying aetiologies in individuals who test negative.

## Methods

### Design and setting

This prospective observational study on young adults with pharyngotonsillitis in Swedish primary health care was a renewed analysis of data collected by Hedin et al. in a prospective aetiological case–control study of pharyngotonsillitis [14], and by Pallon et al. in a subsequent follow-up study [16]. While the previous studies compared aetiological findings between patients and asymptomatic controls, the current study focused on the clinical signs and symptoms of different aetiologies in patients. However, the controls still played a small part in this study, as they were used to calculate aetiological predictive values (see “Statistical analyses”).

The study took part in Kronoberg County in the south of Sweden, which during the study period had a population of approximately 190,000, or about 2% of the Swedish population. To serve this population were two hospitals and 34 primary health care centres (PHCC), five of which participated in the study [14]. The participating PHCCs were located in urban areas and were chosen by convenience.

### Participants

Patients aged 15–45 years who presented to the phone triage nurse with an acute sore throat as a major complaint and who were sufficiently ill to motivate a doctor’s visit according to national guidelines [10], were asked to participate. The national guidelines advise that patients with compelling signs of viral infection should neither be tested for GAS nor treated with antibiotics; that only patients with 3–4 Centor criteria should be tested for GAS; and that patients with severe symptoms or immunosuppression should always be examined by a doctor [10]. If the doctor interpreted the symptoms as infectious pharyngotonsillitis, the patient was recruited after signing a form for informed consent. Asymptomatic controls were recruited from patients 15–45 years old who belonged to the same primary health care centre and consulted for non-infectious causes. We aimed for a consecutive sampling of all eligible patients, but ended up with a convenience sample as it was hard to engage all nurses and doctors in recruitment. The intended ratio of patients to controls was one (see “Statistical analyses”), but turned out closer to two; neither did we manage to fully match the controls in age and sex with the patients.

### Data collection

We asked the doctors to approach each participant as they would normally do, with the addition of completing a form with data about background characteristics, signs and symptoms, diagnosis, tests and treatment.

### Microbiological procedures

As previously described [14], all patients and controls were sampled from the nasopharynx, throat and blood and screened with routine culture for β-haemolytic streptococci (Lancefield group A, C, and G); with anaerobic culture for *Fusobacterium necrophorum*; with serology for Epstein–Barr virus; with single PCR for Influenza A and B viruses and *Mycoplasma pneumoniae;* and with multiplex real-time PCR for two intracellular bacteria and 13 viruses: *M.* *pneumoniae*, *Chlamydophila pneumoniae*, Adenovirus, Bocavirus, Coronavirus NL63, Coronavirus OC43, Coronavirus HKU1, Coronavirus 229E, Enterovirus, Influenza A virus, Influenza B virus, Metapneumovirus, Parainfluenzavirus, Rhinovirus and Respiratory syncytial virus. The primers and probes used in the multiplex PCR have been described elsewhere [17].

RADTs for GAS are routinely used at most Swedish primary health care centres. The only RADT kit available in Region Kronoberg during the study period was QuickVue Dipstick Strep A (Quidel Corporation, San Diego, CA, USA), a lateral-flow immunoassay using antibody-labelled particles. The test detects either viable or nonviable organisms directly from throat swabs.

### Follow-up

We reviewed all electronic medical records from the primary health care and hospitals for the 3 months following inclusion, to see if the patients had made any reconsultations for a sore throat, for a complication—defined here as sinusitis, peritonsillitis, media otitis, mastoiditis, lymphadenitis, necrotizing fasciitis, meningitis, sepsis, glomerulonephritis or rheumatic fever—or for tonsillectomy (ICD-codes for the studied outcomes are provided in Additional file 1: Table S1).

### Statistical analyses

Data was analysed using SPSS 23.0 software (IBM, Armonk, NY, USA) and MedCalc (MedCalc Software Ltd, Ostend, Belgium). Due to non-normal distribution and small sample sizes continuous variables were reported as median (interquartile range [IQR]). Confidence intervals for sensitivity and specificity were calculated using the binomial (Clopper–Pearson) “exact” method. Confidence intervals for positive and negative predictive values were calculated as standard logit confidence intervals according to Mercaldo et al. [18]. Receiver operating characteristic (ROC) curves with area under the curve (AUC) were calculated to evaluate the diagnostic performance of a RADT for GAS at different levels of Centor score. For comparison of independent categorical data, we used two-sided Pearson χ^2^-test, Fisher’s exact test and Mantel–Haenzel trend test. p-values < 0.05 were considered as significant. Multiple logistic regression was used to predict aetiology from signs and symptoms: in the crude model, univariate odds ratios (OR) with 95% confidence intervals were calculated for each sign and symptom, using the “Enter” method; in the multiple model, adjusted odds ratios (aOR) were calculated with 95% confidence intervals. To ensure that there would be at least ten participants per variable in the multiple model, the variables were limited to the four Centor criteria (no cough, lymphadenitis, fever, and tonsillar coating), age and rapid attendance (duration ≤ 3 days). “No coryza” was excluded from the model due to collinearity with “no cough”. Univariate ORs for Centor score 1 through 4 were calculated with logistic regression using Centor 0 as reference category.

This study is based on clinical data previously collected in conjunction with an aetiological case–control study [14], where the intended sample size of 150 patients and 150 controls was primarily chosen so that each participant would represent a percentage larger than one. Moreover, this sample size was also calculated to be able to detect a 10% difference in the prevalence of *F. necrophorum* between patients and controls with a power of 0.8 and an ɑ value of 0.05 [14], which was hypothesized from a small pilot study by one of the authors (MS), where *F. necrophorum* was detected in 11% of patients and 5% of controls (unpublished data). Due to the small numbers of single pathogens, we created mutually exclusive groups before analysis: “only viruses”, “only bacteria”, “viruses and bacteria”, and “no pathogen”. In addition, we grouped all patients with a bacterial finding into “any bacteria”, and all patients with GAS positive culture into “GAS”. A Centor score [8] for each patient was calculated by adding one point each for absence of cough, temperature ≥ 38.5 °C, cervical lymphadenitis and tonsillar coating (for a maximum score of 4).

As there exists no reference standard to determine if a throat infection is caused by GAS or if the detection rather represents a GAS colonisation with a concomitant viral infection, regular predictive values only indicate the presence of GAS, not the presence of disease. *Aetiological predictive value*, on the other hand, is a statistical method that adjusts for asymptomatic carriage when interpreting an aetiological test [19], and it provides positive and negative predictive values with 95% confidence intervals. The requisites for a calculation are: (1) the prevalence of the pathogen among both patients and asymptomatic individuals, (2) the sensitivity of the test, and (3) the “theta” value—the ratio of GAS prevalence in asymptomatic individuals and in patients with a sore throat caused by another pathogen. Based on previous work [19], we assumed a 90% sensitivity of throat culture to detect GAS and a theta value of 0.9.

## Results

### Characteristics of patients and controls

We included 220 patients with a median age of 33 (range 15–48). Their characteristics are presented in Table 1. To be able to calculate aetiological predictive values we also included 126 controls, with a median age of 31 (range 16–46). The controls differed from the patients in having a higher proportion of women (76%) and a lower proportion with frequent episodes of a sore throat (7%). A full table of characteristics of patients and controls has been published elsewhere [14], but is also provided in Additional file 2: Table S2.

**Table 1:** Clinical signs and symptoms of different aetiologies in patients with a sore throat, number (%) if not otherwise stated

		Mutually exclusive groups					
Clinical signs and symptoms	Total n = 220	Only viruses n = 52	Only bacteria n = 85	Viruses + bacteria n = 18	No pathogen n = 65	GAS n = 66	Any bacteria n = 103
Age (years), median (IQR)	33 (23–39)	28 (21–38)	34 (24–40)	35 (26–38)	32 (23–40)	36 (33–40)	34 (26–39)
Female	141/220 (64)	34 (65)^a^	58 (68)^a^	8 (44)	41 (63)^a^	43 (65)	66 (64)
Smoker	30/215 (14)	7 (13)^a^	11 (13)^a^	2 (11)	10 (15)^a^	5 (8)	13 (13)
Days with symptoms, median (IQR)	4 (3–7)	4 (3–7)	3 (3–5)	3 (3–4)	6 (3–10)	3 (3–4)	3 (3–5)
Longstanding sore throat before inclusion	69/215 (32)	15 (29)^a^	22 (26)^a^	4 (22)	28 (43)^a^	16 (24)	26 (25)
Frequent sore throats	72/216 (33)	24 (46)^a,^^†^	24 (28)^a^	6 (33)	18 (28)^a^	18 (27)	30 (29)
Tonsillectomised	29/219 (13)	8 (15)^a^	11 (13)^a^	2 (11)	8 (12)^a^	11 (17)	13 (13)
Antibiotics last month	17/216 (8)	6 (12)^a^	7 (8)^a^	0 (0)	4 (6)^a^	3 (5)	7 (7)
Coryza	89/220 (40)	35 (67)^†††^	20 (24)	7 (39)	27 (42)	12 (18)	27 (26)
Cough	88/220 (40)	35 (67)^†††^	18 (21)	9 (50)	26 (40)	12 (18)	27 (26)
Temperature ≥ 38.5 °C	128/215 (60)	32 (62)^a^	56 (66)^a^	13 (72)	27 (42)^a^	48 (73)	69 (67)
Lymphadenitis	130/215 (60)	32 (62)^a^	56 (66)^a^	10 (56)	32 (49)^a^	44 (67)	66 (64)
Tonsillar coating	84/207 (41)	15 (29)^a,††^	45 (53)^a^	7 (39)	17 (26)^a^	36 (55)	52 (50)
Palatal petechiae	25/207 (12)	9 (17)	10 (12)	3 (17)	3 (5)	8 (12)	13 (13)
Duration ≤ 3 days	96/213 (45)	21 (40)	43 (51)	10 (56)	22 (34)	36 (55)	53 (51)
Centor 0	16/220 (7)^a^	5 (10)^a^	3 (4)^a^	1 (6)	7 (11)^a^	2 (3)	4 (4)
Centor 1	50/220 (23)^a^	14 (27)^a^	8 (9)^a^	4 (22)	24 (37)^a^	6 (9)	12 (12)
Centor 2	69/220 (31)^a^	20 (38)^a^	26 (31)^a^	5 (28)	18 (28)^a^	16 (24)	31 (30)
Centor 3	54/220 (25)^a^	10 (19)^a^	28 (33)^a^	7 (39)	9 (14)^a^	24 (36)	35 (34)
Centor 4	31/220 (14)^a^	3 (6)^a^	20 (24)^a^	1 (6)	7 (11)^a^	18 (27)	21 (20)
Centor 3–4	85/220 (39)	13 (25)^†††^	48 (56)	8 (44)	16 (25)	42 (64)	56 (54)

*GAS* group A streptococci

^†^p = 0.03 compared to “only bacteria”

^††^p = 0.006 compared to “only bacteria”

^†††^p < 0.001 compared to “only bacteria”

^a^These numbers were previously published by Hedin et al. [14] but are republished here for the sake of completeness

### Detected aetiology

The microbial findings in patients and controls were previously reported by Hedin et al. [14]. In summary, 155/220 patients (71%) had at least one of the 20 targeted microorganisms. Bacteria were found in 103 patients (47%) and viruses in 70 patients (32%). GAS was the most common finding (66 patients; 30%). Among controls, 3/126 (2.4%) had GAS and 17/126 (13%) had a bacterial finding.

### Clinical signs and symptoms

Table 1 presents the frequencies of clinical signs and symptoms in different aetiological groups. Cough and coryza were more common in patients with only viruses compared to patients with only bacteria, as was a history of frequent sore throats. Tonsillar coating was more common in those with only bacteria, as was a Centor score of 3–4. Prevalence of fever, lymphadenitis, petechiae and seeing a doctor within 3 days were similar between the two groups. Patients with no detected pathogen waited the longest before seeing a doctor, with a median of 6 days of symptoms prior to the visit. They also more commonly reported a sore throat lasting a long time compared to the other groups (p = 0.02). GAS comprised the majority of bacterial findings, with the frequencies of this group resembling those of “only bacteria” and “any bacteria”.

Among the 85 patients with a Centor score of 3–4, bacteria were found in 56 (66%), and any microorganism was found in 69 (81%). Thus, bacteria were detected in 56/69 (81%) patients with a microbial finding. Clinical signs and symptoms of the 85 patients with a Centor score of 3–4, grouped by “only viruses”, “only bacteria”, “viruses + bacteria”, and “no pathogen” are presented in Additional file 3: Table S3. Among the 16 (19%) patients with “no pathogen”, the frequencies of signs and symptoms resembled those of “only bacteria” most closely.

### Predictive values of clinical findings

Odds ratios and predictive values for GAS and any bacterial findings are presented in Table 2. In the multiple logistic regression model, tonsillar coating and absence of a cough were significantly associated both with GAS and any bacterial findings, whereas fever and lymphadenitis were not.

**Table 2:** Odds ratios and predictive values for the presence of group A streptococci or any bacterial finding among patients with a sore throat (n = 220)

Clinical findings	Group A streptococci (n = 66)					Any bacteria (n = 103)				
	OR (95% CI)	aOR, model 1 (95% CI)	PPV (95% CI)	NPV (95% CI)	EPV (95% CI)	OR (95% CI)	aOR, model 1 (95% CI)	PPV (95% CI)	NPV (95% CI)	EPV (95% CI)
No coryza	2.8 (1.5–5.4)^††^	–	41 (37–46)	87 (79–92)	97 (88–100)	3.2 (1.8–5.6)^†††^	–	58 (52–63)	70 (61–77)	90 (75–97)
No cough	4.4 (2.2–8.8)^†††^	6.0 (2.5–14)^†††^	41 (36–46)	86 (79–92)	97 (88–100)	3.1 (1.7–5.4)^†††^	2.7 (1.4–5.1)^††^	58 (52–63)	69 (61–77)	90 (74–97)
Temperature ≥ 38.5 °C	2.7 (1.4–5.1)^††^	1.4 (0.60–3.1)	38 (33–43)	79 (71–86)	96 (86–100)	2.1 (1.2–3.7)^††^	1.5 (0.76–2.8)	54 (48–59)	61 (53–69)	88 (70–97)
Lymphadenitis	1.7 (0.90–3.1)	1.6 (0.71–3.4)	34 (29–39)	74 (66–81)	96 (83–100)	1.6 (0.93–2.8)	1.2 (0.65–2.3)	51 (45–56)	56 (48–64)	86 (65–96)
Tonsillar coating	3.1 (1.7–5.8)^†††^	3.5 (1.5–8.0)^††^	43 (35–51)	76 (70–81)	97 (88–100)	3.0 (1.7–5.4)^†††^	2.5 (1.3–4.7)^††^	62 (53–70)	59 (53–64)	92 (77–98)
Palatal petechiae	1.2 (0.48–2.9)	–	32 (18–51)	68 (66–70)	95 (54–100)	1.4 (0.59–3.1)	–	52 (34–69)	51 (48–53)	87 (41–98)
Duration ≤ 3 days	1.9 (1.1–3.5)^†^	1.4 (0.65–3.0)	38 (31–45)	74 (68–80)	96 (85–100)	1.9 (1.10–3.3)^†^	1.3 (0.69–2.5)	55 (48–62)	57 (51–63)	89 (69–97)
Age, years	1.09 (1.05–1.13)^†††^	1.12 (1.07–1.18)^†††^	–	–	–	1.02 (0.99–1.05)	1.02 (0.99–1.06)	–	–	–
Centor 3–4	4.5 (2.4–8.3)^†††^	–	49 (42–57)	82 (77–87)	98 (91–100)	3.6 (2.0–6.4)^†††^	–	66 (57–74)	65 (60–70)	94 (81–99)
Centor 3–4 + positive RADT	5.8 (1.4–24)^†^	–	93 (81–97)	97 (84–100)	–	–	–	–	–	–
Centor 0^a^	1	–	13 (3–38)	69 (67–70)		1	–	25 (10–50)	51 (50–53)	
Centor 1	0.96 (0.17–5.3)	–	12 (6–23)	65 (62–68)		0.95 (0.26–3.5)	–	24 (15–36)	47 (43–50)	
Centor 2	2.1 (0.43–10)	–	23 (16–33)	67 (63–71)		2.4 (0.72–8.3)	–	45 (36–55)	52 (48–57)	
Centor 3	5.6 (1.2–27)^†^	–	44 (34–56)	75 (71–78)		5.5 (1.6–20)^††^	–	65 (53–75)	59 (55–63)	
Centor 4	9.7 (1.9–50)^††^	–	58 (42–73)	75 (72–77)		6.3 (1.6–25)^††^	–	68 (51–81)	57 (54–59)	

Crude (univariate) odds ratio (OR) and adjusted ORs (aOR) from the logistic regression are presented with 95% confidence intervals. The multiple model adjusts for the four Centor criteria (no cough, lymphadenitis, fever, and tonsillar coating), age and duration ≤ 3 days. “No coryza” was excluded from the model due to collinearity with “no cough”

*PPV* positive predictive value (true positives/all positives), *NPV* negative predictive value (true negatives/all negatives), *EPV* positive aetiological predictive value, *95% CI* 95% confidence interval, *RADT *rapid antigen detection test

^†^p < 0.05; ^††^p < 0.01; ^†††^p < 0.001

^a^Odds ratios for Centor score 1 through 4 are calculated with Centor 0 as reference category

The positive predictive values were low to moderate for single symptoms, and generally were better at predicting any bacteria than GAS specifically. The negative predictive values were the highest for absence of a cough and absence of coryza, indicating that a finding of cough or coryza would rule out most cases of GAS.

A regression analysis of the Centor score with 0 as reference category revealed a positive association between odds ratios for GAS and any bacteria and increasing score, which was mirrored in the predictive values. Again, the analysis showed a better prediction of any bacteria than of GAS. Adding the result of RADT for GAS to patients with Centor 3–4 increased the positive predictive value from 49 to 93% (Table 2).

### Aetiological predictive values

As the carriage rate of GAS in controls was only 3/126 (2.4%), compared to 30% in symptomatic patients, the Aetiological predictive value of a positive culture for GAS reached 95% (95% CI 81–100), implying an infection in most detected cases. The carriage rate of any bacteria was however higher (13%), resulting in an Aetiological predictive value of any bacterial finding in culture or PCR that was somewhat lower: 84% (95% CI 62–95). Aetiological predictive values for single symptoms in addition to a sore throat were higher than for a sore throat alone, as presented in Table 2.

### Performance of the rapid antigen detection test

**Fig. 1:**
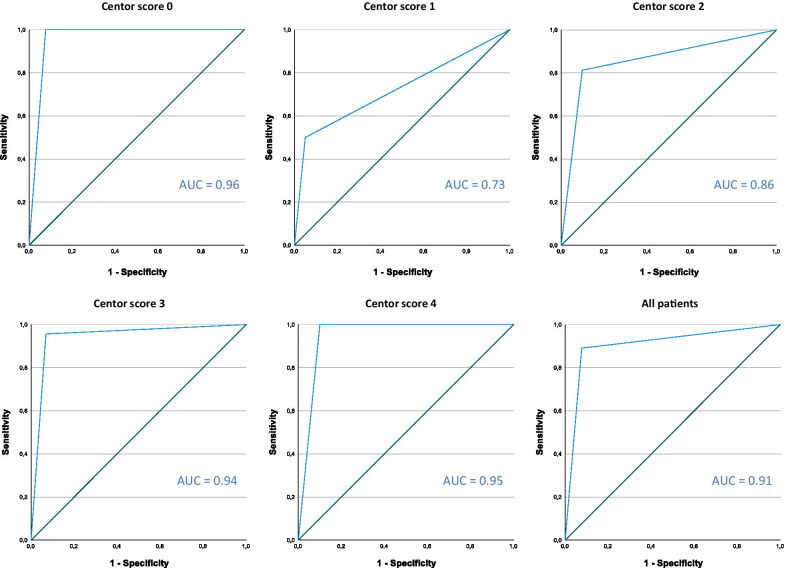
ROC-curves of a rapid antigen detection test (RADT) for group A streptococci (GAS) at different Centor scores, with throat culture as reference. *AUC* area under the curve. Confidence intervals for the AUC values are given in Table 3

In total, 207/220 patients (94%) had an RADT for GAS, despite the test not being a mandatory part of the original study protocol. The 13 patients not tested were evenly distributed with regard to Centor score. Table 3 shows sensitivity, specificity, predictive values, and area under the curve of the test based on our data set, and Fig. 1 displays the ROC-curves. Both sensitivity and positive predictive values increased with higher Centor scores, whereas the negative predictive values were overall high. Of the 37 test negative patients with Centor score 3–4, the underlying aetiology was “any bacteria” in 14 cases (38%), “only viruses” in 12 cases (32%) and “no pathogen” in 11 cases (30%). The detected bacteria were *F. necrophorum* (n = 9), group G streptococci (n = 3), group C streptococci (n = 2), and GAS (n = 1).

**Table 3:** Sensitivity, specificity and predictive values for a rapid antigen detection test (RADT) for group A streptococci (GAS) at different Centor scores

Centor score	All patients (n = 220)		Patients tested with RADT (n = 207)						
	n (%)	Prevalence of GAS in culture n (%)	Tested n (%)	Positive n (%)	Sensitivity^a^ (95% CI)	Specificity^a^ (95% CI)	PPV^a^ (95% CI)	NPV^a^ (95% CI)	AUC^a^ (95% CI)
0	16 (7)	2 (13)	15 (94)	3 (20)	100 (16–100)	92 (64–100)	67 (23–93)	100	0.96 (0.86–1.0)
1	50 (23)	6 (12)	46 (92)	5 (11)	50 (12–88)	95 (83–99)	60 (24–88)	93 (85–97)	0.73 (0.46–0.99)
2	69 (31)	16 (23)	67 (97)	18 (27)	81 (54–96)	90 (79–97)	72 (52–86)	94 (85–98)	0.86 (0.74–0.98)
3	54 (25)	24 (44)	52 (96)	24 (46)	96 (78–100)	93 (77–99)	92 (74–98)	96 (80–99)	0.94 (0.87–1.0)
4	31 (14)	18 (58)	27 (87)	18 (67)	100 (80–100)	90 (56–100)	94 (73–99)	100	0.95 (0.84–1.0)
Total	220 (100)	66 (30)	207 (94)	68 (33)	89 (79–95)	92 (87–96)	84 (74–90)	95 (90–97)	0.91 (0.86–0.96)

*RADT* rapid antigen detection test, *GAS* group A streptococci, *PPV* positive predictive value (true positives/all positives), *NPV* negative predictive value (true negatives/all negatives), *AUC* area under the curve, *95% CI* 95% confidence interval

^a^All numbers for RADT are calculated with throat culture as reference standard for the detection of group A streptococci

### Follow-up

A total of 207 patients (94%) could be followed up. Of these, 21 (10%) reconsulted for a sore throat within 30 days, 17 of whom had a new episode and 4 had non-resolving symptoms. One patient (0.5%) had a complication (sinusitis). Patients with Centor score 4 reconsulted most frequently (5/27; 19%), in contrast to Centor score 0, where none did that. A trend test, however, showed no evidence of a positive association between score and reconsultation (Mantel–Haenzel p = 0.16). A multiple logistic regression model adjusted for covariates, revealed no association between reconsultation and absence of a cough, temperature ≥ 38.5 °C, cervical lymphadenitis, tonsillar coating, or antibiotic prescription (data not shown).

After 3 months a total of 32 patients (16%) had reconsulted for a sore throat. Again, the highest proportion was among patients with Centor score 4 (7/27; 26%) (Mantel–Haenzel p = 0.054).

## Discussion

### Principal findings

In this prospective observational study on young adults visiting primary health care with pharyngotonsillitis, we reanalysed data from Hedin et al. [14] from a more clinical perspective, to study how various clinical signs and symptoms could predict the detected aetiologies. In addition, we followed the patients for three months to analyse any associations between the clinical presentation at inclusion and subsequent reconsultation for a sore throat or a complication.

No single sign or symptom was sufficiently useful to rule in bacteria or viruses, and combining them into a Centor score of 3–4 only modestly raised the positive predictive values, to 49% for GAS and 66% for any bacterial finding. Cough and coryza were rare in patients with GAS and had a negative predictive value of 86%, making these symptoms useful to rule out this pathogen. Aetiological predictive values were high for both GAS and any bacterial finding, meaning that a positive finding represents a true infection rather than carriage in most cases [19]. The RADT was excellent at ruling out GAS regardless of Centor score, whereas the positive predictive value was only acceptable for patients with Centor 3–4. We found no evidence of an association between increasing Centor score and reconsultation within 3 months.

### Strengths and weaknesses

Though some of these results were previously published [14, 16], those articles aimed to describe the clinical characteristics of different pathogens. In this study we attempted to shift the focus to a clinician’s perspective, examining how any given sign and symptom in a patient can predict the presence or absence of different viruses and bacteria. The use of both culture and PCR has enabled us to classify the aetiology as viral or bacterial with greater certainty than with culture alone. In addition, we made no assumptions of aetiology in patients with no detected pathogen, but instead grouped them separately. A limitation of the renewed analysis, however, was that the small size of the study forced us to analyse most microorganisms grouped instead of individually.

Though we could not include all consecutive patients due to busy offices, and though the summer season was excluded, the study interfered minimally in the everyday clinical management of the patients, and thus mirrors typical patients and conditions in Swedish primary health care. This is also the case for the evaluation of signs and symptoms, which is partly a subjective task.

Registering details from the clinical management made it possible to describe the performance of the RADT for GAS. As this test was not asked for, we gave no specific instructions on sampling technique to the participating centres, but RADTs are routinely used in Swedish primary care, and both doctors and laboratory staff are trained in the sampling procedure; it thus mirrors everyday clinical practise.

Other strengths of the study were the sampling of asymptomatic controls, which enabled us to measure the presence of bacteria and viruses and calculate aetiological predictive values; registering individual signs and symptoms rather than the total Centor score; and the prospective approach, which enabled us to follow patients over time.

Pharyngotonsillitis is more common in children than in adults [20, 21], but the prevalence of bacterial pathogens differs with age [22, 23] and we therefore found it reasonable to focus on children and adults separately. At the time of this study we had already started to plan such a project on paediatric sore throat.

### Interpretation

Though many symptoms of pharyngotonsillitis require very large sample sizes to discriminate between GAS and non-GAS aetiology [4], cough and coryza are generally considered viral features [2], and our study also found these symptoms more frequently in patients with microbiological analyses positive for only viruses than in patients with only bacteria. On the other hand, tonsillar coating was more frequent in patients where only bacteria were found. The regression analysis found tonsillar coating and absence of cough to be significantly associated with both findings of GAS and any bacteria, which is in line with a large meta-analysis that showed “any exudates” to have the strongest discriminatory power for GAS [4]. As presented by others [4, 9, 24], no single sign or symptom, however, reached sufficiently high positive predictive values to diagnose GAS or any bacterial finding with certainty. However, both cough and coryza, which are often found together, had a negative predictive value for GAS > 85%, making these symptoms useful for ruling out this pathogen, though not any bacterial findings.

Combining single symptoms into Centor score increased the predictive values for both GAS and any bacterial findings. The positive predictive value increased with every point, and at Centor score 3–4 the positive predictive value was greater than for any single symptom. However, only patients with a score of 4 had a probability for GAS that was greater than the probability of not having GAS. In line with previous reports [11, 13], Centor score was better at predicting any bacterial findings than GAS alone, which is explained by high scores also in many patients with group C or G streptococci or *F. necrophorum*.

The negative predictive value of Centor score 3–4 for GAS was modest, and for any bacterial findings even lower. Furthermore, low Centor scores were not very predictive of viruses.

Adding more items to a score could increase the predictive values, but at the cost of usefulness. The comprehensive nine-item score of Joachim et al. [25], for instance, was created to diagnose GAS in low-resource settings, but is hard to remember.

To our surprise, almost a fifth of the patients with Centor score 3–4 had no detected pathogen, though an infectious aetiology rather than non-infectious seems more likely at these levels. This absence of pathogens might be explained by errors made during sampling, handling, transportation, or analysis [26]. Although one can only speculate about the underlying aetiology, 81% of the patients with a Centor score of 3–4 and a detected aetiology had a bacterial finding, and the frequencies of clinical signs and symptoms in patients with “no pathogen” most closely resembled those of patients with “only bacteria” (Additional file 3: Table S3).

The problem with insufficient precision of clinical scores in diagnosing GAS can be overcome with rapid antigen detection tests, which have great sensitivity and specificity [27]. Several guidelines recommend such a test [2, 3, 10], but it should be restricted to patients with Centor score 3–4 as this is the only group shown to benefit from antibiotics [28]. Another reason to restrict testing to these patients, which was apparent in our study, is that both sensitivity and specificity of the RADT increase with Centor score [29], leading to false positives and false negatives in patients with Centor score 0–2. The large number of patients with low scores in this study, together with a lower sensitivity of the RADT, reduced the positive predictive values, which were only 60–70% at these levels.

The negative predictive values of RADT were high at all levels of Centor score, in line with previous reports [27], and this shows that a negative test result rule out most cases of GAS. Correctly used, an RADT could therefore lower the antibiotic prescription rate to half [2, 30, 31]. On the other hand, a too liberal use of the test at lower scores, as was the case in our study group, will encourage antibiotic treatment in patients with no apparent benefit, and this could contribute to medicalisation and changed expectations among patients [12]. The fact that an overwhelming majority of the patients were tested is a major deviation from National guidelines [10], and deserves a study on its own with regard to doctor’s attitudes.

If group C and G streptococci and *F. necrophorum* are considered important pathogens, the RADT will miss them, whereas both the Centor score and the FeverPAIN score will detect many of them [11–13, 15]. It then becomes a question of which bacteria to treat [12, 13, 15]. Little et al. [12] showed that basing antibiotic treatment on an RADT for GAS did not improve the outcomes regarding pain and time to recovery, compared to using the FeverPAIN score, which, in essence, is a comparison of treating only GAS with treating any bacteria. However, before we have stronger evidence for the benefits of treating other bacteria than GAS, the clinical scores may lead to antibiotic overuse [27].

A commonly overlooked problem in aetiological diagnosis is the possibility of asymptomatic carriage, especially in children [32]. This applies not only to GAS, but also to other streptococci and *F. necrophorum*, and occludes the meaning of a positive test [19]. To correctly assess a finding, one must therefore adjust for the carriage rate. The aetiological predictive value [19] does exactly that, with the assumption that the carriage rate is the same in symptomatic patients and symptomatic controls. In our study, we found a low carriage rate of both GAS and other bacteria, implying that most detected bacteria were responsible for the symptoms, and that aetiological diagnosis is thus meaningful.

The follow-up revealed no strong evidence for an association between individual or combined signs and symptoms and reconsultation, adjusted for antibiotic treatment. This was in line with a previous study, that only found previous medical problems, sex, temperature and muscle aches to be independently but weakly associated with reconsultation [33]. Signs and symptoms thus seem to be inadequate as predictors of future visits for a sore throat, and the clinician should rather focus on other factors that seem to have a greater impact on the tendency to consult for respiratory infections, such as young age, female gender, anxiety, and perceived threats [34]. This could be accomplished by promoting self-management to targeted groups of patients, and providing broader information, such as leaflets and public campaigns [34].

## Conclusions

Signs and symptoms, both single and combined, were insufficient to diagnose GAS or other pathogens; a greater use may instead lie in ruling out GAS, as cough and coryza both exhibited great NPVs. The Centor score was more predictive of any bacterial finding than of GAS, which indicates an overlapping clinical presentation of many bacteria. The RADT was excellent at ruling out GAS regardless of Centor score, whereas the PPV for GAS was only acceptable for patients with a Centor score of 3–4. The low carriage rate of bacteria among asymptomatic controls implied that most detections in patients represented a true infection.

## Supplementary Information

**Additional file 1: Table S1.** List of ICD-codes for outcomes in the follow-up study of 220 patients with a sore throat in primary health care.

**Additional file 2: Table S2.** Characteristics of 220 patients 15–45 years old with acute sore throat in primary health care, and 126 asymptomatic controls 15–45 years old.

**Additional file 3: Table S3.** Clinical signs and symptoms of different aetiologies in 85 patients with a sore throat and a Centor score of 3–4, number (%).

## Data Availability

The data sets generated and analysed during the current study are not publicly available due to Swedish legislation (the Personal Data Act) but are available from the corresponding author on reasonable request.

## Abbreviations

aOR: Adjusted odds ratio
GAS: Group A streptococci
EPV: Positive aetiological predictive value
IQR: Interquartile range
NPV: Negative predictive value
OR: Odds ratio
PCR: Polymerase chain reaction
PPV: Positive predictive value
RADT: Rapid antigen detection test
ROC: Receiver operating characteristic
