# Supplementary material for: The aetiology of pharyngotonsillitis in primary health care: a prospective observational study
Source: BMC Infect Dis. 2021 Sep 17;21:971. doi: 10.1186/s12879-021-06665-9 (PMC8446737; doi:10.1186/s12879-021-06665-9)
Supplement: Supplementary file 1 — Additional file 1: Table S1. List of ICD-codes for outcomes in the follow-up study of 220 patients with a sore throat in primary health care. [file 12879_2021_6665_MOESM1_ESM.docx]

Table S1. List of ICD-codes for outcomes in the follow-up study of 220 patients with a sore throat in primary health care.

| **Description of data** | | | |
| --- | --- | --- | --- |
| The column “Diagnosis code” shows ICD-10 codes. | | | |
| The column “Description” is the standard English description for clarity (not used in Sweden). | | | |
| The column “Diagnosis code, primary care” shows the standard Swedish primary care codes according to a simplified version of ICD-10 (KSH-97). | | | |
| The column “Description, primary health care” is the standard English description for clarity (not used in Sweden). | | | |
| The heading "Intervention code" refers to the national Classification of Health Interventions, or "KVÅ" ("Klassifikation av vårdåtgärder"). | | | |
|  |  |  |  |
| **Diagnosis code** | **Description** | **Diagnosis code, primary healthcare** | **Description, primary health care** |
| J02- | Acute pharyngitis | J02- | Acute pharyngitis |
| J020 | Streptococcal pharyngitis | J02- | Acute pharyngitis |
| J028 | Acute pharyngitis due to other specified organisms | J02- | Acute pharyngitis |
| J029 | Acute pharyngitis, unspecified | J02- | Acute pharyngitis |
| J03- | Acute tonsillitis | J03- | Acute tonsillitis |
| J030 | Streptococcal tonsillitis | J03- | Acute tonsillitis |
| J038 | Acute tonsillitis due to other specified organisms | J03- | Acute tonsillitis |
| J039 | Acute tonsillitis, unspecified | J03- | Acute tonsillitis |
|  |  |  |  |
| J36- | Peritonsillar abscess | J36- | Peritonsillar abscess |
| J369 | Peritonsillar abscess | J36- | Peritonsillar abscess |
|  |  |  |  |
| J01- | Acute sinusitis | J01- | Acute sinusitis |
| J010 | Acute maxillary sinusitis | J01- | Acute sinusitis |
| J011 | Acute frontal sinusitis | J01- | Acute sinusitis |
| J012 | Acute ethmoidal sinusitis | J01- | Acute sinusitis |
| J014 | Acute pansinusitis | J01- | Acute sinusitis |
| J018 | Other acute sinusitis | J01- | Acute sinusitis |
| J019 | Acute sinusitis, unspecified | J01- | Acute sinusitis |
|  |  |  |  |
| H660 | Acute suppurative otitis media | H660 | Acute suppurative otitis media |
| H664 | Suppurative otitis media, unspecified | H669P | Otitis media, unspecified |
| H669 | Otitis media, unspecified | H669P | Otitis media, unspecified |
| H669P | Otitis media, unspecified | H669P | Otitis media, unspecified |
|  |  |  |  |
| H70- | Mastoiditis and related conditions | H70- | Mastoiditis |
| H700 | Acute mastoiditis | H70- | Mastoiditis |
| H701 | Chronic mastoiditis | H70- | Mastoiditis |
| H709 | Mastoiditis, unspecified | H70- | Mastoiditis |
| H750 | Mastoiditis in infectious and parasitic diseases classified elsewhere | H939P | Other disorders of ear |
|  |  |  |  |
| L04- | Acute lymphadenitis | L04- | Acute lymphadenitis |
| L040 | Acute lymphadenitis of face, head and neck | L04- | Acute lymphadenitis |
| L048 | Acute lymphadenitis of other sites | L04- | Acute lymphadenitis |
| L049 | Acute lymphadenitis, unspecified | L04- | Acute lymphadenitis |
|  |  |  |  |
| A41-P | Other sepsis | A41-P | Septicaemia |
| A419 | Sepsis, unspecified | A41-P | Septicaemia |
| A418 | Other specified sepsis | A41-P | Septicaemia |
| A400 | Sepsis due to streptococcus, group A | A41-P | Septicaemia |
| A414 | Sepsis due to streptococcus, group A | A41-P | Septicaemia |
| A408 | Other streptococcal sepsis | A41-P | Septicaemia |
| A409 | Streptococcal sepsis, unspecified | A41-P | Septicaemia |
|  |  |  |  |
| G00- | Bacterial meningitis, not elsewhere classified | Meningitis | G03-P |
| G01- | Meningitis in bacterial diseases classified elsewhere | Encephalitis, myelitis and encephalomyelitis | G04-P |
| G02- | Meningitis in other infectious and parasitic diseases classified elsewhere | Encephalitis, myelitis and encephalomyelitis | G04-P |
| G03- | Meningitis due to other and unspecified causes | Meningitis | G03-P |
| G04- | Encephalitis, myelitis and encephalomyelitis | Encephalitis, myelitis and encephalomyelitis | G04-P |
|  |  |  |  |
| M726 | Necrotizing fasciitis | M799P | Other soft tissue disorders |
|  |  |  |  |
| N008 | Acute nephritic syndrome with other morphologic changes | N05-P | Glomerulonephritis, unspecified |
|  |  |  |  |
| I00- | Acute rheumatic fever without heart disease | I38-P | Acute and chronic endocarditis |
| I01- | Acute rheumatic fever with heart disease | I38-P | Acute and chronic endocarditis |
| I02- | Rheumatic chorea | I38-P | Acute and chronic endocarditis |
|  |  |  |  |
|  |  |  |  |
|  |  |  |  |
|  |  |  |  |
| **Intervention code** |  |  |  |
| EMB10 | Tonsillectomy |  |  |
