# Supplementary material for: The aetiology of pharyngotonsillitis in primary health care: a prospective observational study
Source: BMC Infect Dis. 2021 Sep 17;21:971. doi: 10.1186/s12879-021-06665-9 (PMC8446737; doi:10.1186/s12879-021-06665-9)
Supplement: Supplementary file 2 — Additional file 2: Table S2. Characteristics of 220 patients 15–45 years old with acute sore throat in primary health care, and 126 asymptomatic controls 15–45 years old. [file 12879_2021_6665_MOESM2_ESM.docx]

Table S2. Characteristics of 220 patients 15–45 years old with acute sore throat in primary health care, and 126 asymptomatic controls 15–45 years old.

|  | Patients | Control group | Χ^2^ |
| --- | --- | --- | --- |
|  | n=220 | n=126 | p |
| Age, median (range) | 15–48 | 16–46 |  |
| Female (%) | 64 | 76 | 0.02 |
| Smoker (%) | 14 | 8.1 | 0.1 |
| A history of often having a sore throat (%) | 33 | 7.3 | <0.001 |
| Previous tonsillectomy (%) | 13 | 12 | 0.8 |
| Antibiotic treatment in the last month (%) | 7.9 | 5.6 | 0.4 |

This table was previously published by Hedin et al. [12].
