# Supplementary material for: The aetiology of pharyngotonsillitis in primary health care: a prospective observational study
Source: BMC Infect Dis. 2021 Sep 17;21:971. doi: 10.1186/s12879-021-06665-9 (PMC8446737; doi:10.1186/s12879-021-06665-9)
Supplement: Supplementary file 3 — Additional file 3: Table S3. Clinical signs and symptoms of different aetiologies in 85 patients with a sore throat and a Centor score of 3–4, number (%). [file 12879_2021_6665_MOESM3_ESM.docx]

Table S3. Clinical signs and symptoms of different aetiologies in 85 patients with a sore throat and a Centor score of 3–4, number (%).

|  | Only viruses | Only bacteria | Viruses + bacteria | No pathogen |
| --- | --- | --- | --- | --- |
|  | n=13 | n=48 | n=8 | n=16 |
| Coryza | 5 (39) | 12 (25) | 3 (38) | 5 (31) |
| Cough | 4 (31) | 3 (6) | 2 (25) | 2 (13) |
| Temperature ≥38.5°C | 13 (100) | 43 (90) | 13 (100) | 16 (100) |
| Lymphadenitis | 13 (100) | 41 (85) | 6 (75) | 13 (81) |
| Tonsillar coating | 7 (54) | 35 (73) | 6 (75) | 12 (75) |
